# Identification of blood at simulated crime scenes using silver nanoparticles with SERS

**DOI:** 10.55730/1300-0527.3687

**Published:** 2024-07-14

**Authors:** Uğur KÖROĞLU, Necdet SAĞLAM, Uğur TAMER, Ramazan AKÇAN, İsmail Hakkı BOYACI, Eylül EVRAN

**Affiliations:** 1 Department of Nanotechnology and Nanomedicine, Graduate School of Science and Engineering, Hacettepe University, Ankara, Turkiye; 2 Department of Analytical Chemistry, Faculty of Pharmacy, Gazi University, Ankara, Turkiye; 3 Micro-Electro-Mechanical Systems (MEMS) Center, Middle East Technical University, Ankara, Turkiye; 4 Department of Forensic Medicine, Faculty of Medicine, Hacettepe University, Ankara, Turkiye; 5 Department of Food Engineering, Faculty of Engineering, Hacettepe University, Ankara, Turkiye

**Keywords:** Surface-enhanced Raman spectroscopy, nanoparticle, forensic sciences, bionanotechnology, biochemistry, analytical chemistry

## Abstract

The analysis of substances and samples obtained from a crime scene is very important in solving forensic cases. To determine the variables involved in a crime and to expedite the investigation process, the rapid analysis of body fluids in small quantities and within environments containing diverse components is particularly necessary. For this reason, it is of great importance to analyze biological fluids with rapid, noncontaminating, nondestructive, low-cost, and accurate techniques. In recent years, with advancements in laser technology, spectroscopic methods have been introduced as analytical techniques in forensic medicine and chemical studies. This study focuses on surface-enhanced Raman spectroscopy (SERS) to demonstrate the detection of blood samples in simulated crime scenes. To minimize the background signal from fluorescent biomolecules in blood, dilution was performed with two different components and Raman analysis was performed for four different concentrations of blood. In general, a decrease in noise in the spectra was observed as the blood was diluted. Crime scenes consisting of pure blood, blood diluted with ethanol and distilled water (1:2, 1:4, and 1:8), a blood–mineral water mixture, a blood–cherry juice mixture, and silver nanoparticle-added mixtures were simulated, and their spectra were examined. Chemometric analyses of the data were performed. Despite high noise and low peak intensities, blood-identifying signals were detected when examining different blood concentrations. It was observed that silver nanoparticles provided high enhancement of blood peaks thanks to their strong plasmonic properties.

## 1. Introduction

One of the important roles of forensic science is to obtain appropriate evidence from crime scenes. Materials, samples, and other data that may be evidence collected from the scene are very important [1]. Among a wide variety of substances when it comes to forensic evidence, body fluids (blood, semen, sweat, saliva, urine, etc.) are invaluable [2–4]. By analyzing the data obtained from biological stains, predictions can be made about the form or time of the event. It is very important to determine whether a stain found in the examined area is any organic/biological liquid, including fruit/vegetable juices, or whether it belongs to a living organism and is ultimately a human body fluid, and then to determine its characteristics. Distinguishing between different liquids may be difficult depending on the level of complexity at the scene and how much time has passed [5]. Furthermore, the amount of sample material typically recovered from crime scenes is limited. Even if it is clear what the collected liquid is, a series of analyses must be performed and officially verified. Blood is a body fluid frequently encountered at crime scenes and can be recognized by its color. However, many tests must be done to determine characteristics of the blood and who it belongs to. Most tests designed for the determination of blood only provide basic detection, and after analysis the sample is destroyed and cannot be used for other tests. For this reason, it is very important that the test be performed correctly. If the test is not performed correctly, erroneous results may be obtained and the sample may become unusable. In addition, even if a correct result is obtained, laboratory testing procedures take a long time, causing delays in the implementation of justice. Therefore, the limitations of current methods necessitate a new approach to blood analysis that prioritizes speed, accuracy, and sample preservation [3].

Particularly in the last decade, advancements in laser technology have significantly bolstered the capabilities of spectroscopic methods. These improvements, coupled with the development of novel light detectors, have propelled spectroscopic techniques to the forefront of molecular structure characterization. As a result, surface-enhanced Raman spectroscopy (SERS) and other spectroscopic methods are finding increasing applications in the analysis of bodily fluids [6,7]. Studies that started with clinical research have contributed to forensic science in the analysis of blood and other body fluids. Various studies using SERS for blood identification have been presented [8,9].

SERS utilizes nanoparticles to diminish the background signal and fluorescence generated by the complex molecular makeup of blood while simultaneously amplifying the SERS signal. Thanks to the localized plasmon surface resonances of nanoparticles, Raman scattering intensity increases and the SERS signal becomes stronger. In the realm of SERS, silver nanoparticles stand out due to their ease of synthesis and their remarkable plasmonic characteristics, which significantly enhance SERS signals [10–13].

This study explores the capabilities of SERS for detecting blood in an environment containing a similar liquid. We investigate whether SERS can differentiate between the two liquids despite their close physical properties. Furthermore, this study examines the impact of silver nanoparticles on the SERS signal of blood, focusing on achieving quantitative detection and analyzing how these nanoparticles influence the resulting spectrum.

## 2. Materials and methods

In this investigation, SERS was employed to identify and characterize blood, a common forensic evidence material. To optimize SERS effectiveness, silver nanoparticles were synthesized through established chemical methods and utilized to amplify the signal.

### 2.1. Materials and equipment

Blood was drawn from a male volunteer who was over 18 years of age. Pure cherry juice and mineral water were purchased.

An aluminum thin-film-layer (TLC) plate, silica gel-coated with fluorescent indicator F254, was used as the substrate, purchased from Merck (Darmstadt, Germany). A new thin-film part produced from the same batch was used for each analysis. For silver nanoparticle synthesis, silver nitrate (AgNO_3_) was obtained from Merck (Darmstadt, Germany) and sodium hydroxide (NaOH) and hydroxylamine hydrochloride (NH_2_OH·HCl) were purchased from Sigma-Aldrich (Taufkirchen, Germany).

A Raman spectrophotometer (UVP Portable Raman Spectroscope, 785 nm wavelength; Analytik Jena, Jena, Germany) was used for SERS measurements of biological samples. Nanoparticle characterization was performed with a Specord Ultraviolet-Visible spectrophotometer (Analytik Jena, Jena, Germany) and JEOL 2100 HRTEM (JEOL, Tokyo, Japan). For sample preparation, a deionized water unit (Millipore Simplicity, Darmstadt, Germany), centrifuge device (Eppendorf, Hamburg, Germany), precision balance (Shimadzu, Tokyo, Japan), ultrasonic bath (Bandelin-Sonorex, Berlin, Germany), and micropipettes (1–10, 10–100, and 100–1000 μL) were used.

### 2.2. Statistics and chemometrics

MATLAB software (MathWorks Inc., Natick, MA, USA) and PLS_Toolbox version 8.2 (Eigenvector Research Inc., Manson, WA, USA) were used for the chemometric analysis of data. Principal component analysis (PCA) and partial least squares discriminant analysis (PLS-DA) were applied for discrimination of the blood samples. PLS-DA was utilized with training and test datasets at a 3:1 ratio for discrimination of mixed blood samples and Ag-treated mixed blood samples.

Furthermore, to assess the performance of PLS-DA discrimination, the sensitivity rate (STR, %), specificity rate (SPR, %), and model efficiency rate (EFR, %) were determined using metrics including true positive rate (TPR), false negative rate (FNR), true negative rate (TNR), and false positive rate (FPR).


(1) STR=TPR/(TPR+FNR)

(2) SPR=TNR/(TNR+FPR)

(3) EFR=100-(FPR+FNR)

### 2.3. Synthesis of nanoparticles

To synthesize silver nanoparticles (AgNPs), 3.3 × 10^−3^ M NaOH and 1.68 × 10^−3^ M NH_2_OH·HCl solutions were mixed with a magnetic stirrer in 90 mL of distilled water to prepare a base solution. A 0.01 M AgNO_3_ solution was quickly added to the base solution to initiate the reduction reaction. The clear solution turned a metallic gray color, indicating the formation of AgNPs. The nanoparticles were then purified by centrifugation.

**Figure 1:**
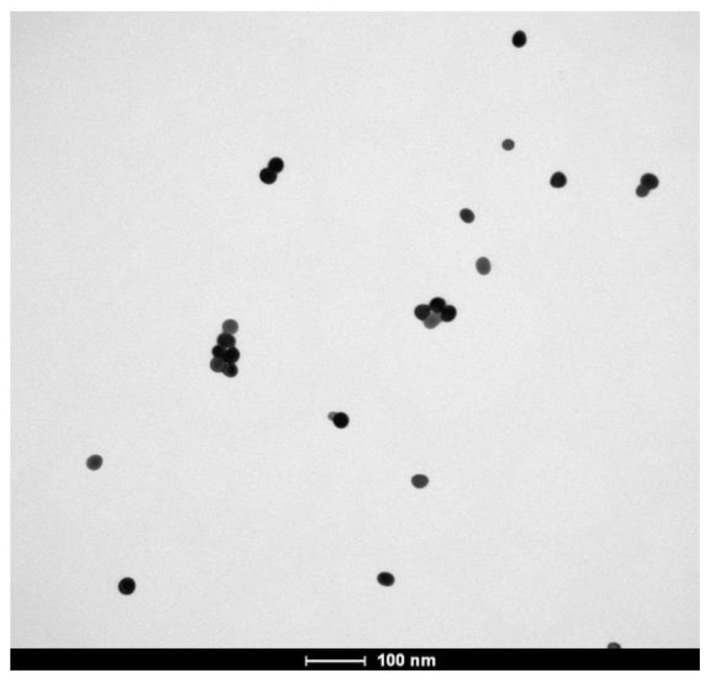
TEM image of silver nanoparticles.

**Figure 2:**
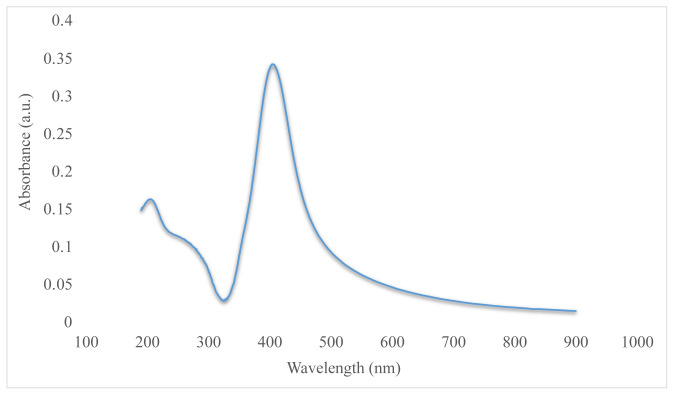
UV-Vis analysis of silver nanoparticles.

The prepared AgNPs were examined under TEM and their UV-Vis absorption spectra were obtained. Judging from the TEM image given in Figure 1, it can be seen that the AgNPs have diameters of approximately 30 nm. In the UV-Vis graph in Figure 2, it can be seen that the absorption spectrum peaked at approximately 400 nm. This study compared the absorption spectra of AgNPs synthesized from AgNO_3_ at various time points. As illustrated in Figure 2, the peak observed at approximately 400 nm aligns with the findings of Bhui et al. [14].

### 2.4. Preparation of samples

The blood taken from the volunteer was first poured into seven different Eppendorf tubes in a volume of 100 μL. Distilled water and ethanol were added to six tubes to dilute the blood. Specifically, 100 μL, 300 μL, and 700 μL of distilled water and ethanol were added to these tubes for blood concentrations of 1/2, 1/4, and 1/8. In the seventh tube, whole blood was stored without changing its concentration.

At the simulated crime scene, 100 μL of blood sample prepared by dilution with distilled water in the previous stage was taken, and 100 μL of mineral water was added to four tubes while 100 μL of cherry juice was added to four tubes.

### 2.5. Test procedure

In the first stage, the blood sample was applied to the substrate surface. After waiting for it to dry for about 2 h, spectra were obtained from 50 different points from a surface area of 1 cm × 1 cm of the spilled liquid. Raman spectroscopy was performed over the range of 500–2000 nm. The noise was suppressed, and baseline correction was performed by applying the BWSpec program to the obtained data. The procedure was repeated for blood concentrations of 1, 1/2, 1/4, and 1/8. Dilution processes were carried out using distilled water and ethanol.

In the second step, 100 μL of cherry juice and 100 μL of blood or 100 μL of mineral water and 100 μL of blood were mixed in eight separate tubes at different blood concentrations. Based on the data obtained in the first step, it was determined that all of these mixtures utilized blood dissolved in distilled water. The mixtures were then added to a surface and allowed to dry for 2 h. Raman spectra were collected for each mixture.

In the third stage, AgNPs were added to the eight mixtures obtained in the second stage. The mixtures were then allowed to dry for 1 h and SERS spectra were examined.

Fifty data points were collected for each sample. The experimental process is presented in Table 1.

## 3. Results and discussion

The SERS technique developed in this study is very promising in the diagnosis and detection of biological fluids. The sensitivity of this method is much higher compared to other spectroscopic techniques such as UV-Vis or Fourier transform infrared (FTIR) analysis. SERS allows the detection of molecules at levels of parts per billion (ppb) and even parts per trillion (ppt). By choosing the right nanoparticle and optimizing the spectroscopic parameters, high selectivity and specificity can be achieved and detection from the fingerprint spectrum becomes much easier. Although the technique used in this study is more expensive than other spectroscopic techniques, its ease of use in the laboratory environment and its ability to be used on the scene make it an important candidate.

The technique used in this study was compared to other methods such as UV-Vis, FTIR, inductively coupled plasma-mass spectrometry (ICP-MS), and high-performance liquid chromatography (HPLC). The new technique stands out thanks to its requirement of a small amount of sample and its fast, specific, and sensitive results. However, considering that the vibrational spectrum alone provides only rough structural information about the analytes, it is anticipated that developing this technique further and combining it with the other methods mentioned above will provide many more advantages [15,16].

With the optimization tests performed, 10× objective and 20 laser power were determined as the spectrum measurement parameters. To improve signal clarity, the baselines of the spectra were adjusted using BWSpec software.

### 3.1. Spectra analysis

**Figure 3:**
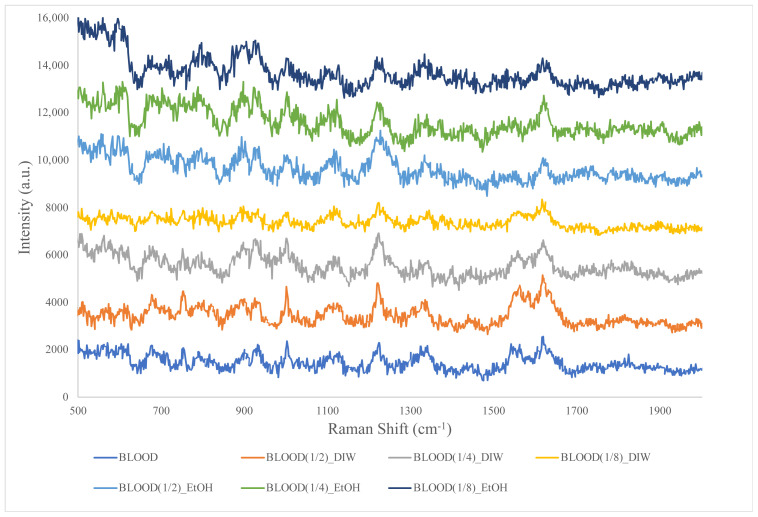
Raman spectrum of blood at different concentrations diluted with ethanol and distilled water.

This study examined blood samples prepared at four different concentrations. These concentrations were achieved by diluting the blood with either distilled water or ethanol. As seen in Figure 3, different reproducible peaks were taken from the TLC surface at 935, 1001, 1221, 1325, 1552, and 1621 cm^−1^ with low signal-to-noise ratios.

Raman analyses performed on different concentrations of blood samples showed that the noise effects were high. However, clear signals of blood were also seen in the spectra. No clear change in signal ratio was detected after dilution with both distilled water and ethanol. As a result of the examinations, it was seen that the same spectra were obtained in both dilution cases. When the blood concentration changed, some blood signals became stronger due to homogeneity, while others gave weaker peaks. The observed spectral features were consistent with findings reported in previous studies [4,8,17–21].

**Figure 4:**
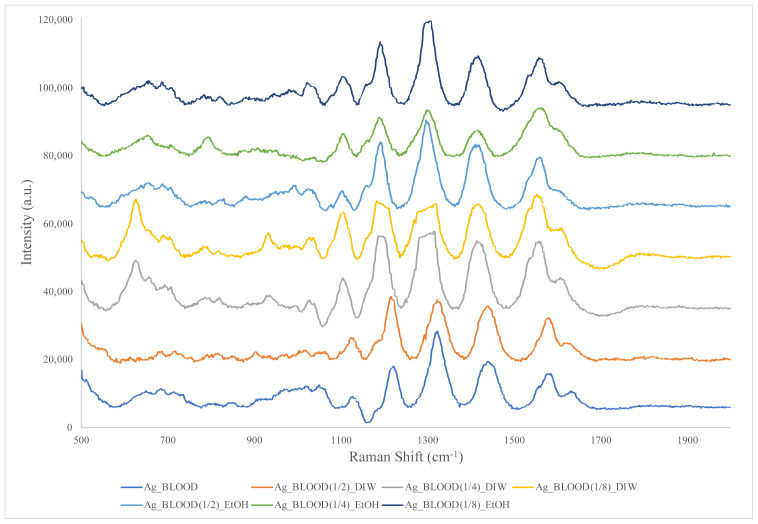
SERS spectra of silver nanoparticle-added blood diluted with ethanol and distilled water.

In the next step, the signal enhancement provided by AgNPs in the SERS spectrum of blood was examined. The spectra of the blood samples with added AgNPs showed many strong bands (Figure 4). Peaks were observed at 791, 935, 1001, 1104, 1190, 1322, 1417, 1551, and 1619 cm^−1^ for blood. Plasmonic effects occurring on the surface of the AgNPs drastically enhanced the Raman scattering signals. Although an increase in the signal level was observed as the blood concentration decreased, widening and distortion of the peaks were detected when the concentration increased to 1/4 and above. This may have been due to the low signal-to-noise ratio that occurred with decreasing concentration, decreased ionization, changes in the environment that generated localized plasmon resonance, aggregation and clumping, or homogeneity problems [22]. It was determined that the data obtained were compatible with the blood spectra taken in the previous stage and those reported in similar studies in the literature [4,8,17–21,23–28].

**Figure 5:**
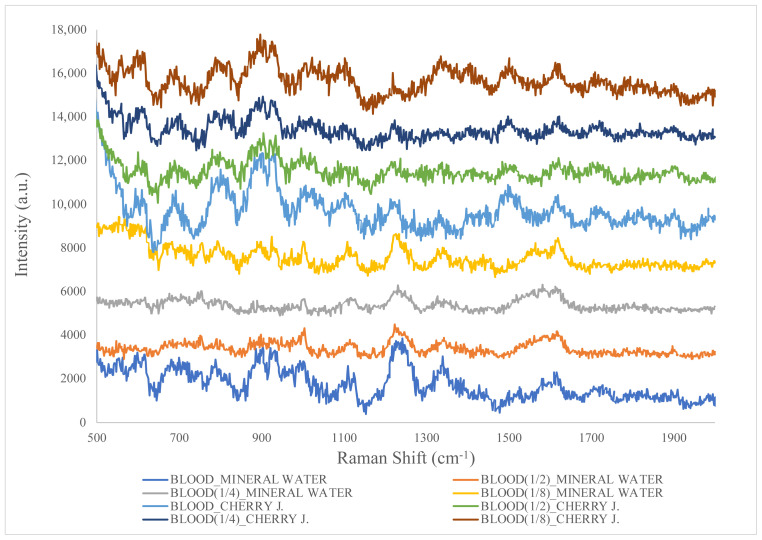
Raman spectra of blood–mineral water and blood–cherry juice mixtures at different concentrations.

Figure 5 shows the Raman spectra of mixtures prepared with mineral water and cherry juice for different concentrations of blood. The spectra obtained from the mixtures gave results similar to those acquired in the presence of blood alone. It was found that the signal-to-noise ratio was low. Although clear spectra could not be obtained due to the noise effect, blood signals were clearly observed. Especially in the simulated crime scenes prepared with mineral water, peak intensities were higher than those of mixtures containing cherry juice. A decrease in signal level was observed with decreasing blood concentration in both simulated crime scenes. When there were fewer analyte molecules, less ionization occurred to activate Raman scattering. This caused the peaks to become less intense. In mixtures prepared with mineral water, blood signals were detected at 789, 937, 1000, 1104, 1221, 1339, 1560, and 1619 cm^−1^. In the mixtures prepared with cherry juice, blood signals were observed at 793, 935, 1000, 1104, 1219, and 1618 cm^−1^. The obtained spectra were compatible with each other in terms of blood peaks [4,8,17–21,23–28].

**Figure 6:**
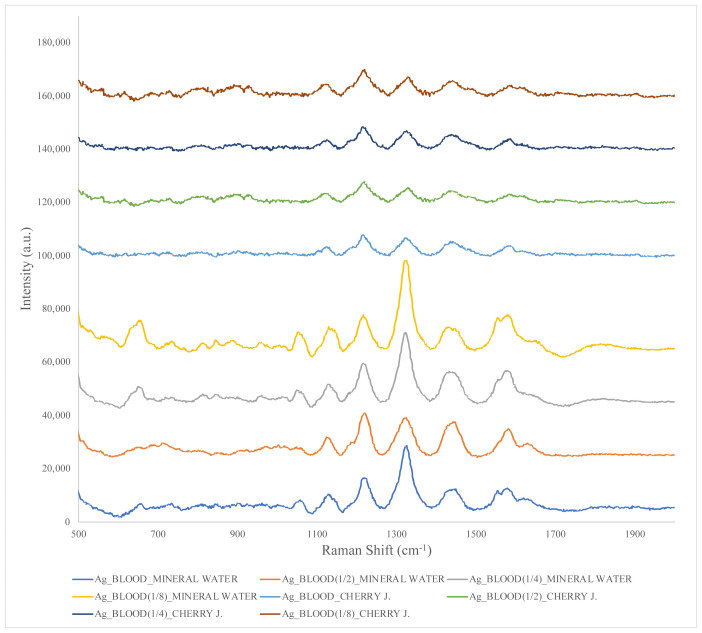
SERS spectra of silver nanoparticle-added blood–mineral water and blood–cherry juice mixtures at different concentrations.

The final step involved the addition of AgNPs to the blood–mineral water and blood–cherry juice samples and SERS spectra were obtained (Figure 6). It was determined that the fluorescence effect was suppressed, the noise levels were minimized, and the spectrum bands belonging to blood were clearly observed in the spectra. When AgNPs were added to simulated crime scenes prepared with blood and mineral water, a large increase in the intensities of SERS spectra was observed in parallel with the dilution of blood, while the same effect was not seen in mixtures prepared with cherry juice. In the previous experimental stage, it was determined that the signal intensities of blood–cherry juice mixtures were lower than those of blood–mineral water mixtures. The difference in signal intensities of blood in the mixtures was mainly due to SERS scattering efficiency, sedimentation and aggregation, and liquid–nanoparticle interactions [20]. Since Raman scattering efficiency depends on the molecular structure and composition of the liquid, liquids with high Raman scattering efficiency increase SERS signal levels more when mixed with blood. It can be stated here that the Raman scattering efficiency of mineral water is higher than that of cherry juice. On the other hand, thanks to the noise effect being suppressed by the addition of AgNPs, the blood spectrum could also be seen in the simulated crime scene prepared with blood and cherry juice.

The SERS spectra of blood–mineral water mixtures to which AgNPs were added gave many strong peaks. Signals at 1055, 1129, 1219, 1329, 1436, 1576, and 1619 cm^−1^ of blood were found in blood–mineral water mixtures. In blood–cherry juice mixtures, blood peaks were observed at 1128, 1219, 1332, 1437, 1569, and 1617 cm^−1^. The spectra were similar to those taken in the previous stages of the research and those reported in similar studies [4,8,17–21,23–28]. The very strong plasmonic properties of silver and its plasmonic properties throughout the entire visible region made the silver stand out [29,30]. In addition, based on the spectra obtained, it seems that it is advantageous to use AgNPs for more accurate analysis. Spectral data showed that at least five distinct peaks were obtained for blood. Raman scattering points obtained for blood were compatible with each other.

### 3.2. PCA analysis

**Figure 7:**
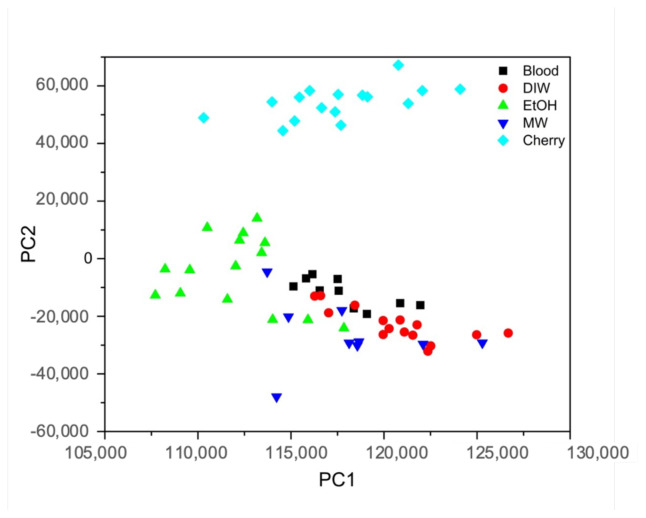
PCA model for discrimination of blood samples: scores on PC1 (90.06%) and PC2 (6.73%).

The PCA method was used to distinguish blood samples. Two different PCA models were developed. Blood samples mixed with deionized water (DIW), ethanol (EtOH), mineral water (MW), and cherry juice were separated using the PCA method. While blood samples mixed with cherry juice and ethanol were distinguished, samples with deionized water, mineral water, and blood alone could not be differentiated (Figure 7).

### 3.3. PLS-DA analysis

**Figure 8:**
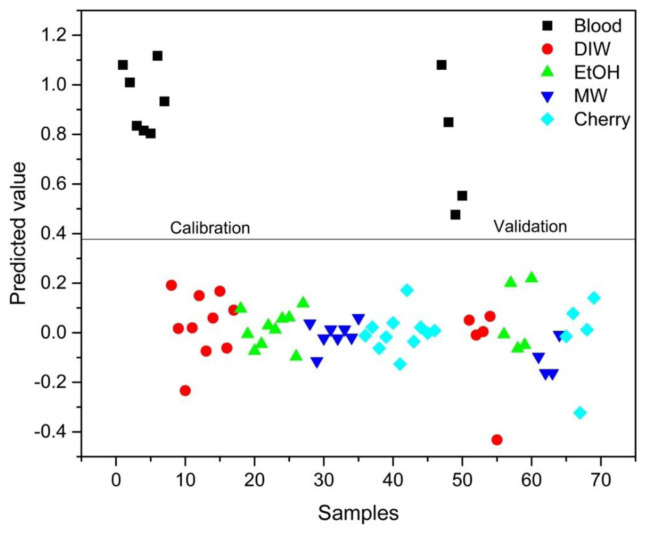
Discrimination of blood from samples mixed with deionized water (DIW), ethanol (EtOH), mineral water (MW), and cherry juice.

**Figure 9:**
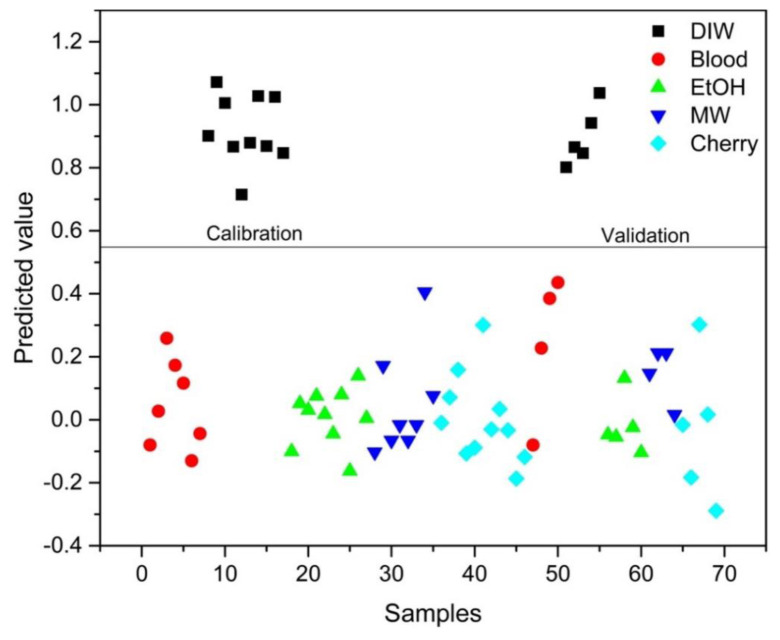
Discrimination of DIW-mixed blood samples from blood and other samples mixed with ethanol (EtOH), mineral water (MW), and cherry juice.

**Figure 10:**
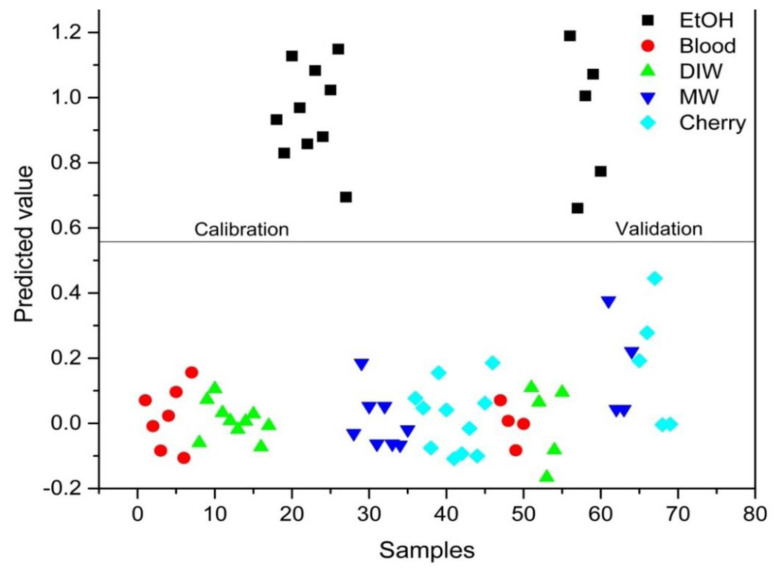
Discrimination of EtOH-mixed blood samples from blood and other samples mixed with deionized water (DIW), mineral water (MW), and cherry juice.

**Figure 11:**
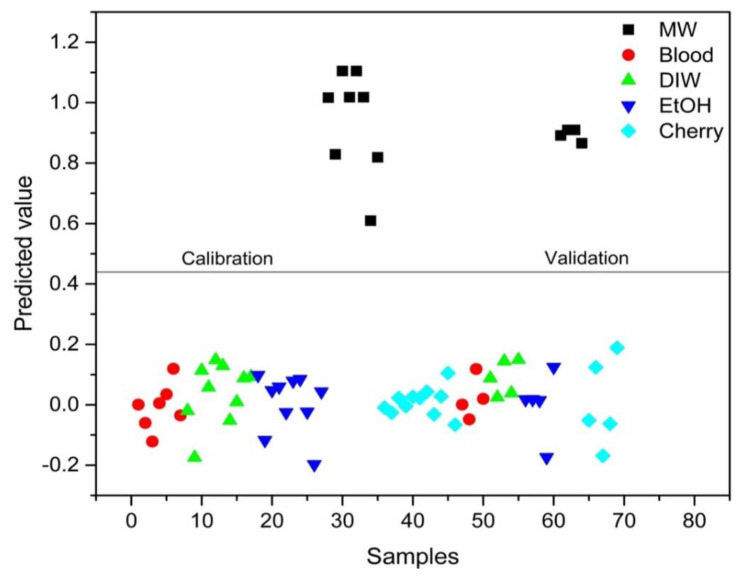
Discrimination of mineral water (MW)-mixed blood samples from blood and other samples mixed with deionized water (DIW), ethanol (EtOH), and cherry juice.

**Figure 12:**
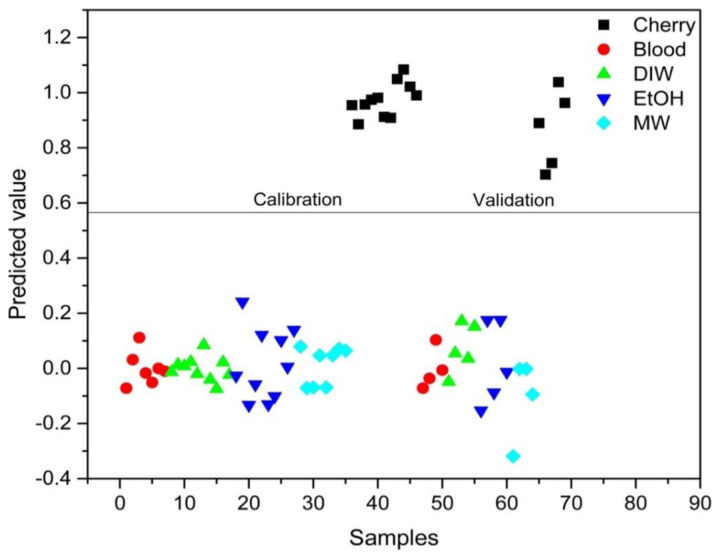
Discrimination of cherry juice-mixed blood samples from blood and other samples mixed with deionized water (DIW), ethanol (EtOH), and mineral water (MW).

**Figure 13:**
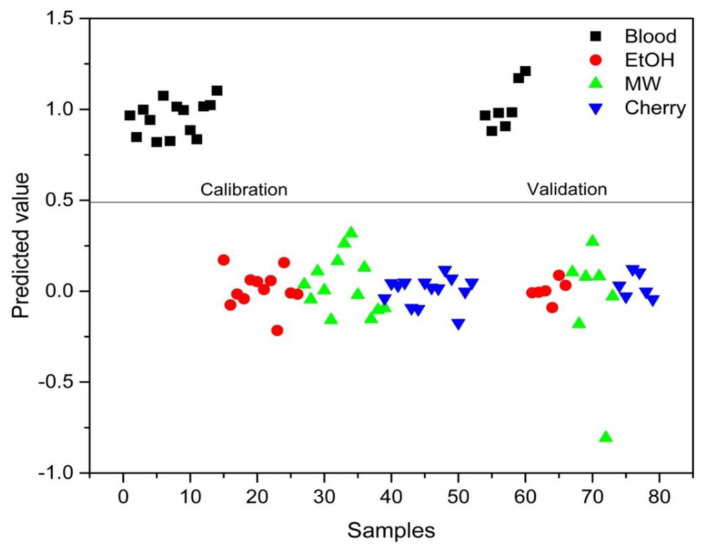
Discrimination of deionized water (DIW) mixed Ag-blood samples from other Ag-blood samples mixed with ethanol (EtOH), mineral water (MW), and cherry juice.

**Figure 14:**
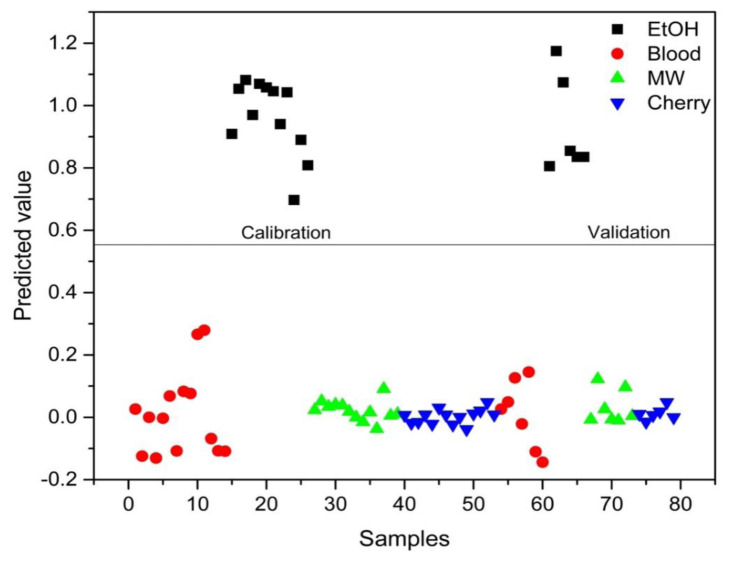
Discrimination of ethanol (EtOH) mixed Ag-blood samples from other Ag-blood samples mixed with deionized water (DIW), mineral water (MW), and cherry juice.

**Figure 15:**
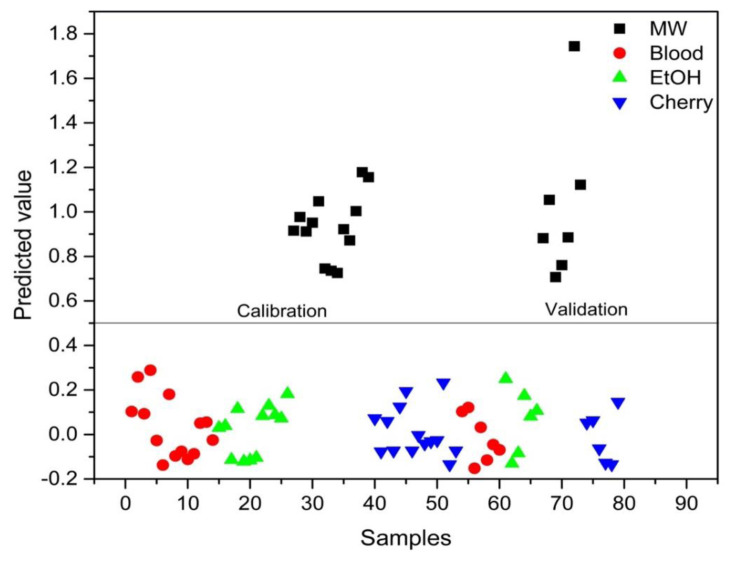
Discrimination of mineral water (MW) mixed Ag-blood samples from other Ag-blood samples mixed with deionized water (DIW), ethanol (EtOH), and cherry juice.

**Figure 16:**
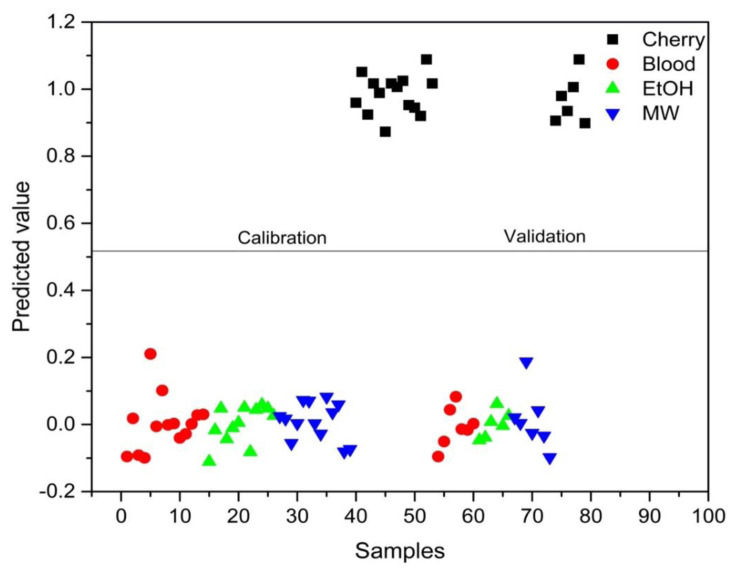
Discrimination of cherry juice mixed Ag-blood samples from other Ag-blood samples mixed with deionized water (DIW), ethanol (EtOH), and mineral water (MW).

PLS-DA analysis was conducted to distinguish between blood alone and mixed samples. Successful discrimination between blood and mixed samples was achieved using PLS-DA. Figures 8–12 show the discrimination of blood, blood–DIW, blood–EtOH, blood–MW, and blood–cherry juice samples, respectively. All separations demonstrated 100% model efficiency, as evidenced in the figures. Figures 13–16 indicate discrimination among Ag-treated blood samples. The performance parameters for all PLS-DA models are presented in Table 2.

Blood samples taken from a crime scene may not always belong to healthy individuals. The person to whom the blood belongs may be a drug addict or a medical patient. In such cases, variations in chemical composition and the presence of unique biomolecules can cause differences in the Raman spectra. This can add another dimension to the research and may require the investigation of other analytical procedures. A study by Nargis et al. published in 2019 showed that the spectrum of a breast cancer patient was different from that of a healthy individual [31]. For this reason, the molecular interactions of the blood signals detected in this study were compiled with findings accumulated from previous studies, as shown in Table 3.

In this study, a fixed substrate and AgNPs produced by a standard procedure were used for the analysis of blood and mixtures. A sample-specific synthesis method was not determined and no substrate selection was made. In particular, the size and shape of the AgNPs directly affected the spectra. In this context, optimizing the nanoparticles to be used will yield better results. It is critical that the device being used has the appropriate features and laser wavelength for the liquids to be analyzed, that it is calibrated, and that it works with the correct parameters. In the analyses, the optimum parameters were determined through test measurements and all spectra were taken while keeping those parameters constant. While preparing the blood and mixtures, no special centrifugation was performed for the samples; the prepared liquids were centrifuged at 14,000 rpm for 5 min before being poured into the TLC plates. In particular, it must be asked to what extent homogeneity was achieved in the prepared simulated crime scenes. Therefore, considering the molecular structure and nature of biological fluids, it would be beneficial to pretreat samples with a standard procedure to obtain reproducible spectral results. In addition, the homogeneous distribution of AgNPs on the surface was ignored.

The findings of this study need further investigation before they can be applied for clinical diagnosis or biomarker discovery. Despite these limitations, this study is an important step in demonstrating that SERS can be used to analyze blood samples at complex crime scenes. Obtaining rapid and precise results in a short period of time is the most important requirement for analyses to be carried out in the field. Considering the possible problems that may occur in the field, using a Raman active surface with added nanoparticles can prevent many problems. In this regard, in future studies, using a direct Raman active substrate, evaluating different nanoparticles for signal enhancement, or comparing different geometries and sizes of AgNPs will contribute to both the creation of a good SERS library and the determination of the necessary substrate for accurate and rapid detection.

## 4. Conclusion

This study has demonstrated the potential of SERS with AgNPs for the rapid and nondestructive identification of blood at crime scenes. Despite some limitations such as noise effects and peak distortion at higher blood concentrations, this method shows promise in providing conclusive results even in complex environments. The addition of AgNPs significantly enhances the SERS signal, improving the detectability of blood even at low concentrations.

Moving forward, further optimization of nanoparticle properties and experimental conditions could enhance the sensitivity and reliability of the method. Additionally, future research should focus on addressing the limitations identified in this study, such as the variability in blood composition and the need for control experiments. With continued refinement and validation, SERS with AgNPs could emerge as a valuable tool in forensic science for the rapid and accurate analysis of biological fluids at crime scenes.

## Figures and Tables

**Table 1:** Experimental procedure.

Stages	Blood concentrations
Spectra of blood diluted with distilled water	1, 1/2, 1/4, 1/8
Spectra of blood diluted with ethanol	1, 1/2, 1/4, 1/8
Spectra after the addition of silver nanoparticles to blood diluted with distilled water	1, 1/2, 1/4, 1/8
Spectra after the addition of silver nanoparticles to blood diluted with ethanol	1, 1/2, 1/4, 1/8
Spectra of a mixture of distilled water–diluted blood and mineral water	1, 1/2, 1/4, 1/8
Spectra of a mixture of distilled water–diluted blood and cherry juice	1, 1/2, 1/4, 1/8
Spectra after the addition of silver nanoparticles to a mixture of distilled water–diluted blood and mineral water	1, 1/2, 1/4, 1/8
Spectra after the addition of silver nanoparticles to a mixture of distilled water–diluted blood and cherry juice	1, 1/2, 1/4, 1/8

**Table 2:** Performance parameters of PLS-DA discrimination of blood and treated samples.

LV	Blood samples	Ag-treated blood samples
	8	9
REMSEC	0.0943344^a^ 0.131244^b^ 0.0995247^c^ 0.102529^d^ 0.0764618^e^	0.110098^f^ 0.0910276^g^ 0.127999^h^ 0.0625064^i^
REMSECV	0.176935^a^ 0.26484^b^ 0.145194^c^ 0.171539^d^ 0.0930372^e^	0.208601^f^ 0.104725^g^ 0.215246^h^ 0.108111^i^
STR (%)	1^a^ 1^b^ 1^c^ 1^d^ 1^e^	1^f^ 1^g^ 1^h^ 1^i^
SPR (%)	1^a^ 1^b^ 1^c^ 1^d^ 1^e^	1^f^ 1^g^ 1^h^ 1^i^
TPR (%)	1^a^ 1^b^ 1^c^ 1^d^ 1^e^	1^f^ 1^g^ 1^h^ 1^i^
FNR (%)	0^a^ 0^b^ 0^c^ 0^d^ 0^e^	0^f^ 0^g^ 0^h^ 0^i^
EFR (%)	100^a^ 100^b^ 100^c^ 100^d^ 100^e^	100^f^ 100^g^ 100^h^ 100^i^

a Blood;

b blood–deionized water;

c blood–EtOH;

d blood–mineral water;

e blood–cherry juice;

f AgNPs–blood;

g AgNPs–blood–EtOH;

h AgNPs–blood–mineral water;

i AgNPs–blood–cherry juice.

**Table 3:** Molecular vibrations of the Raman bands in human blood [17,28,32,33].

Raman shifts of present work (cm^−1^)	Raman shifts of reference works (cm^−1^)	Molecular vibrations and assignments
789–793	791	ν(pyr breathing) Pyrimidine
935–937	935–937	ν_46_ Proline C-C bonds
1000–1001	1000–1003	Phenylalanine
1055	1054	δ(=C_b_H_2_)_asym_
1104	1104	Phenylalanine
1129	1127–1129	ν(C_b_-methyl) ν_5_(C-N)
1190	1190	Thr: CH_3_ rocking
1219–1221	1220–1222	δ(C_m_H) C=N=C stretching
1322–1329	1322	CH_3_CH_2_ twisting
1332	1332	C_5_-O_5_ and C_3_-C_3_ stretching C stretching of phenyl
1339	1338	ν_41_
1373	1371–1374	ν(pyr half-ring)_sym_ ν_4_ T, A, G
1417	1417	C=C stretching
1436–1437	1436–1437	CH_2_
1551–1552	1552	Tryptophan
1560	1560–1563	ν_19_ Tryptophan COO
1569	1566	ν(C_b_C_b_)
1576	1576	Nucleic acid Guanine (N_3_)
1617–1621	1620–1621	ν(C_a_=C_b_)
